# Supplementary material for: Strain maps characterize the symmetry of convergence and extension patterns during zebrafish gastrulation
Source: Sci Rep. 2021 Sep 29;11:19357. doi: 10.1038/s41598-021-98233-z (PMC8481280; doi:10.1038/s41598-021-98233-z)
Supplement: Supplementary file 1 — Supplementary Information 1. [file 41598_2021_98233_MOESM1_ESM.pdf]

Title

Strain maps characterize the symmetry of convergence and extension patterns during Zebrafish gastrulation

Dipanjan Bhattacharya, Jun Zhong, Sahar Tavakoli, Alexandre Kabla, and Paul Matsudaira.

Supplementary Materials:

Movie-1 : The fluorescent images of the embryo development from Dorsal and Ventral views during the time course of our experiment.

Movie-2 : The Strain maps along Medio-lateral (Strain M-L) and Anterior-Posterior (Strain A-P) directions in the dorsal view, during the time course of our experiment.

Movie-3 : The curl maps and Strain-trace maps in the dorsal view, during the time course of our experiment.

Movie-4 : The Strains maps of both control and C59 group along A-P and M-L directions in the dorsal view, during the time course of our experiment.

Movie-5 : The Strain maps of both control and C59 group of curl in the dorsal view, during the time course of our experiment.

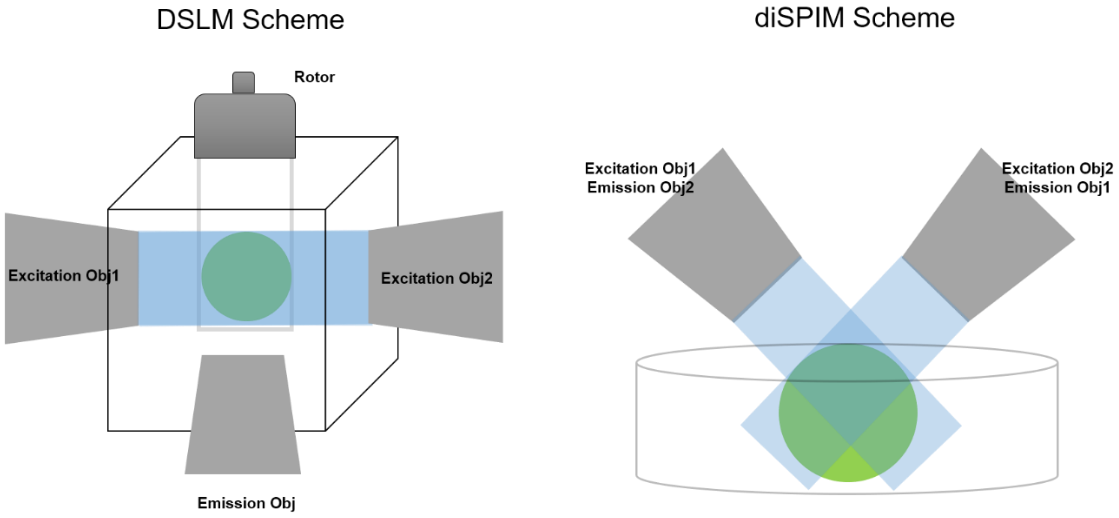

Supplementary Figure 1. Illustration of imaging scheme for DSLM and diSPIM.

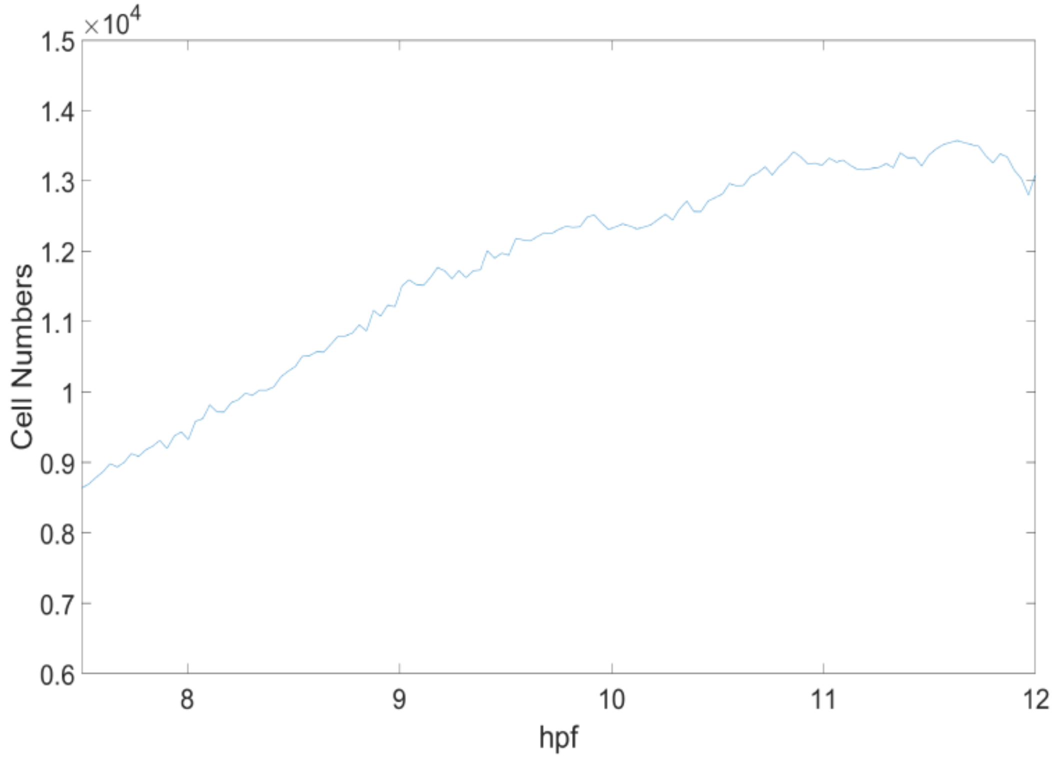

Supplementary Figure 2: The number of cells of the developing embryo during the time-plot of our experiment is plotted here.

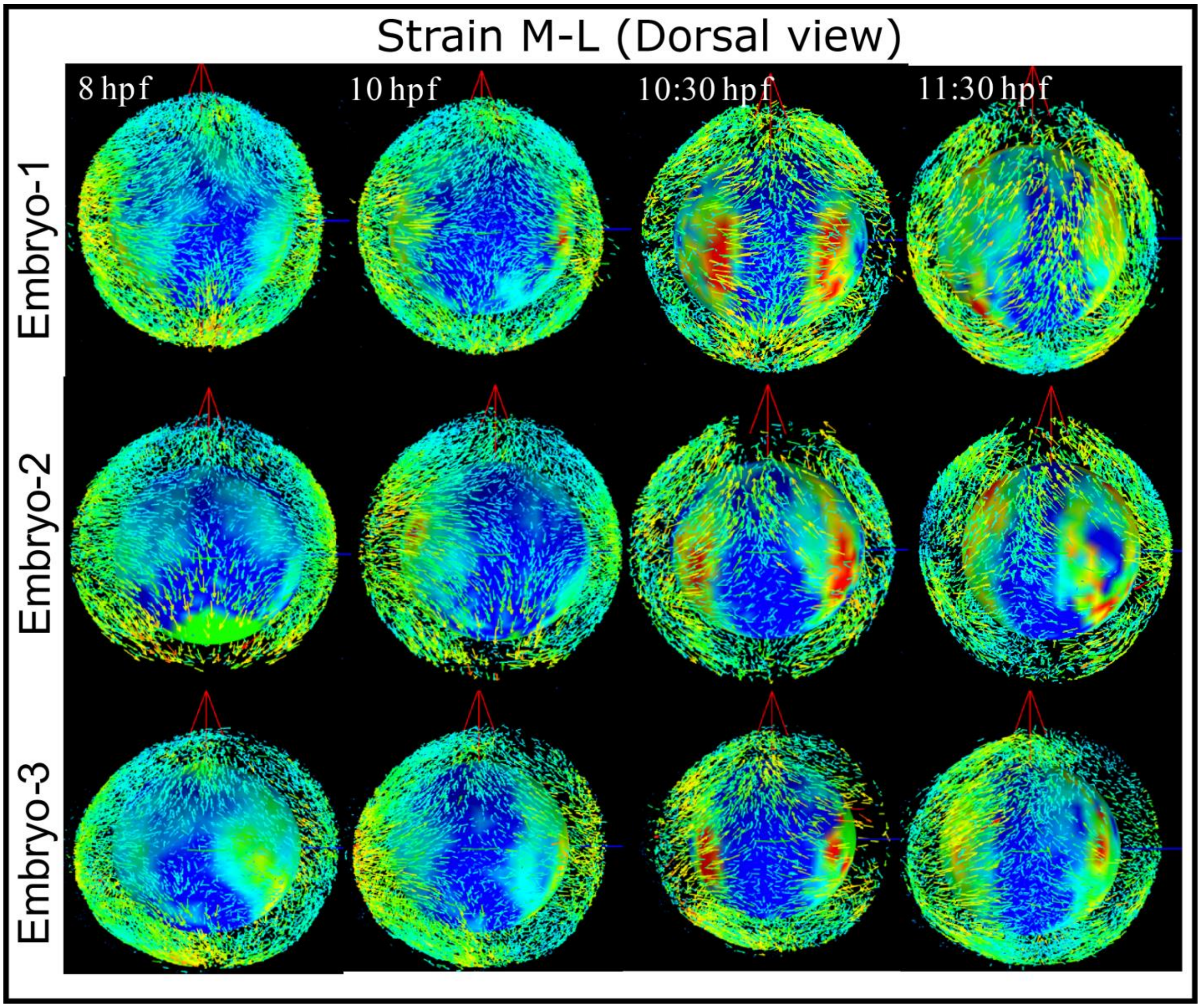

Supplementary Figure 3: The Medio-lateral strain maps (Strain M-L) across different embryos show the same compaction behaviour in the dorsal view, before 100% epiboly. At 30 minutes post 100% epiboly the A-P strain shows a expansion domains in both sides of the body axis.

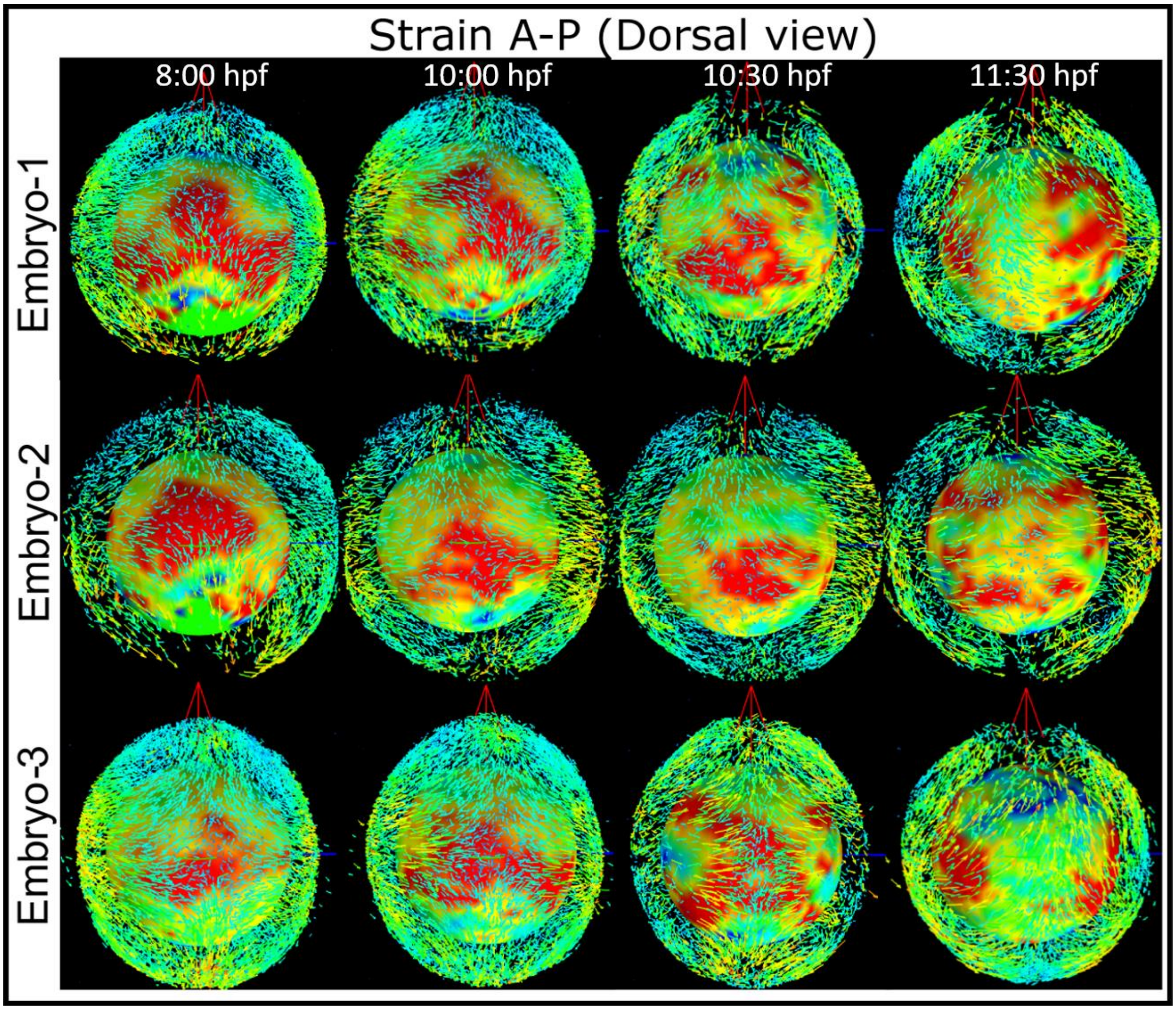

Supplementary Figure 4: The expansion in the Anterior-Posterior strain (Strain A-P) in the dorsal side is conserved across different embryos during the time course of our experiment.

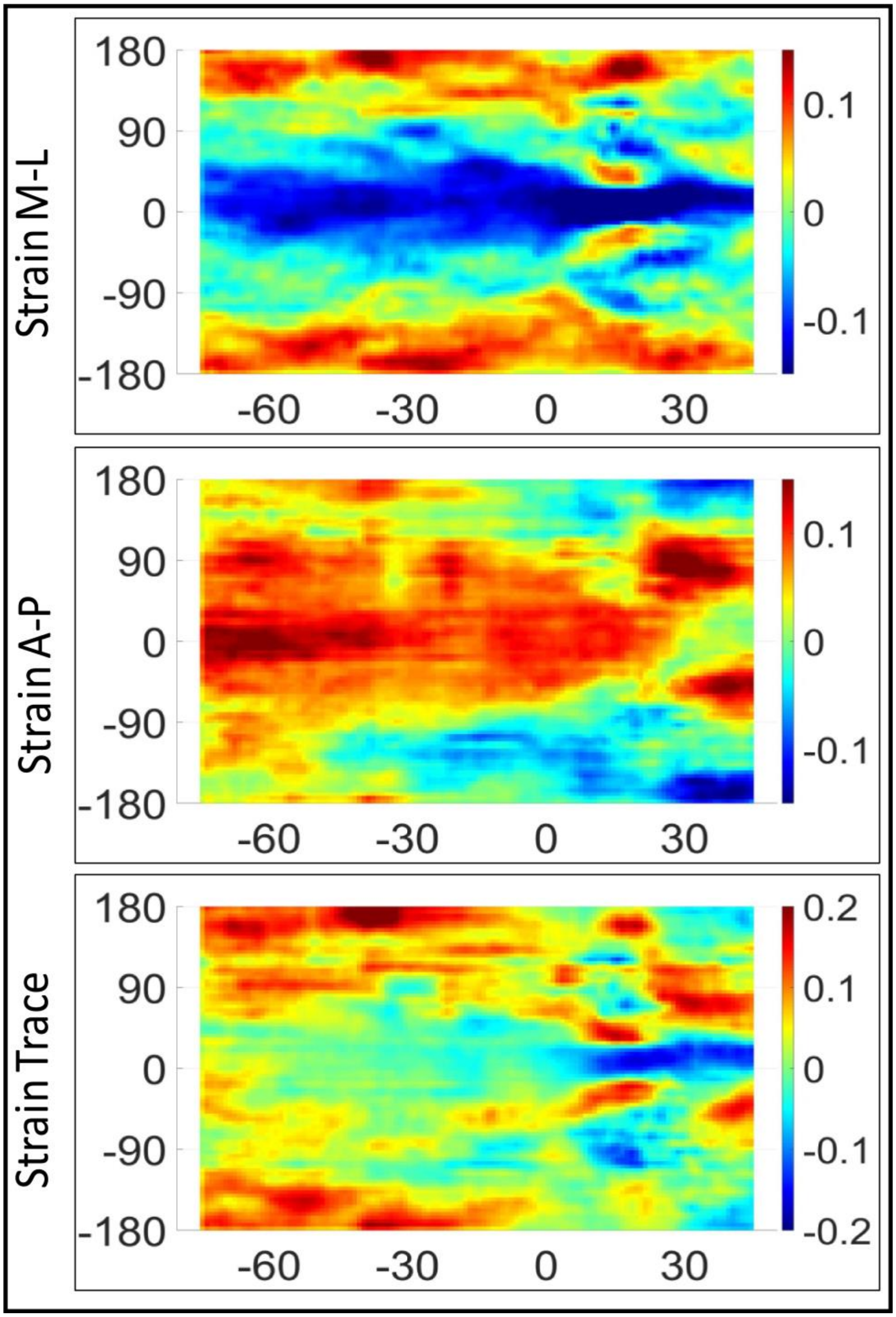

Supplementary Figure 5: The kymograph of Medio-lateral strain (Strain M-L), Anterior-Posterior strain (Strain A-P), and strain trace along the equatorial axis.

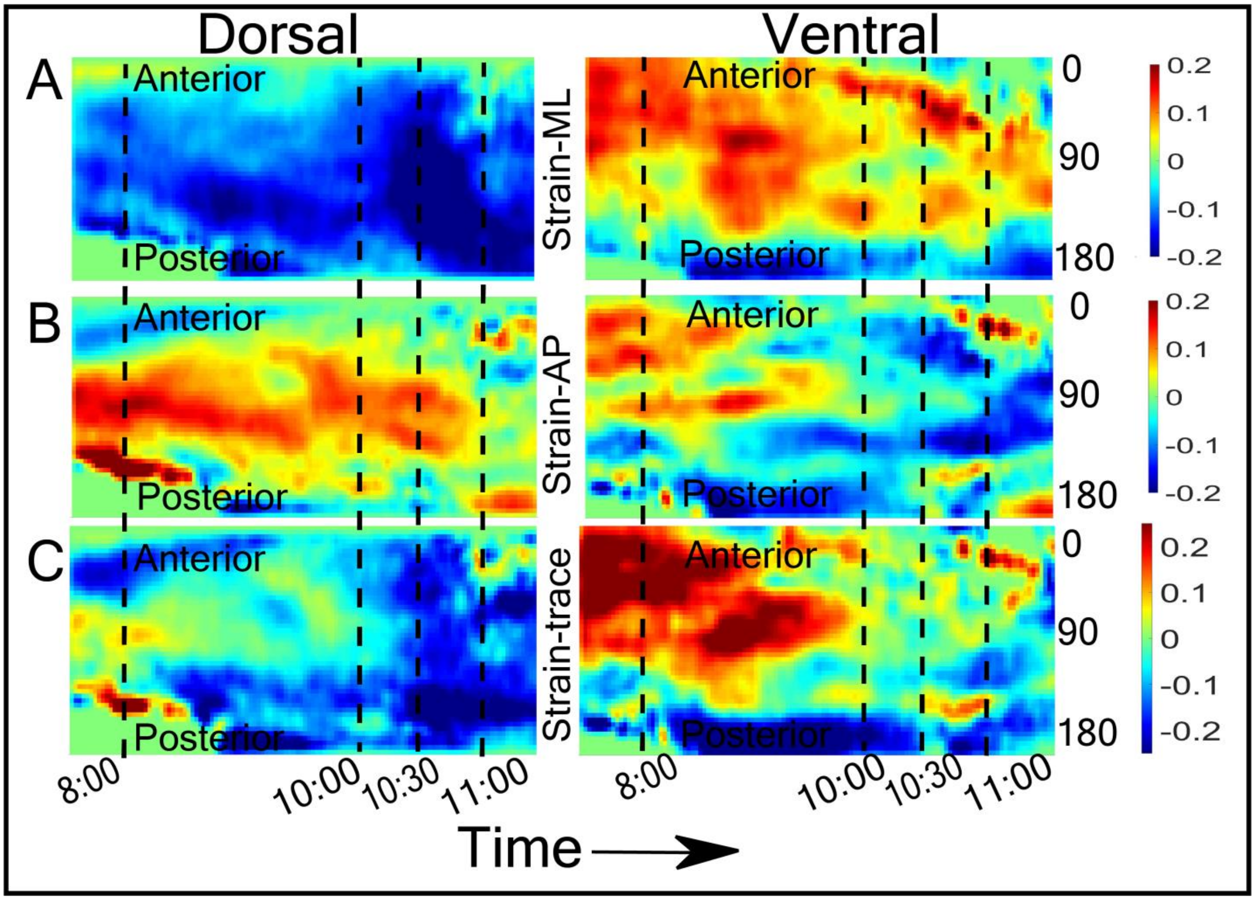

Supplementary Figure 6: The kymograph of Anterior-Posterior strain (Strain A-P), Medio-lateral strain (Strain M-L) and strain trace along body axis in Dorsal view and ventral view respectively. (0° represents Animal Pole, 180° represents the Vegetal Pole).

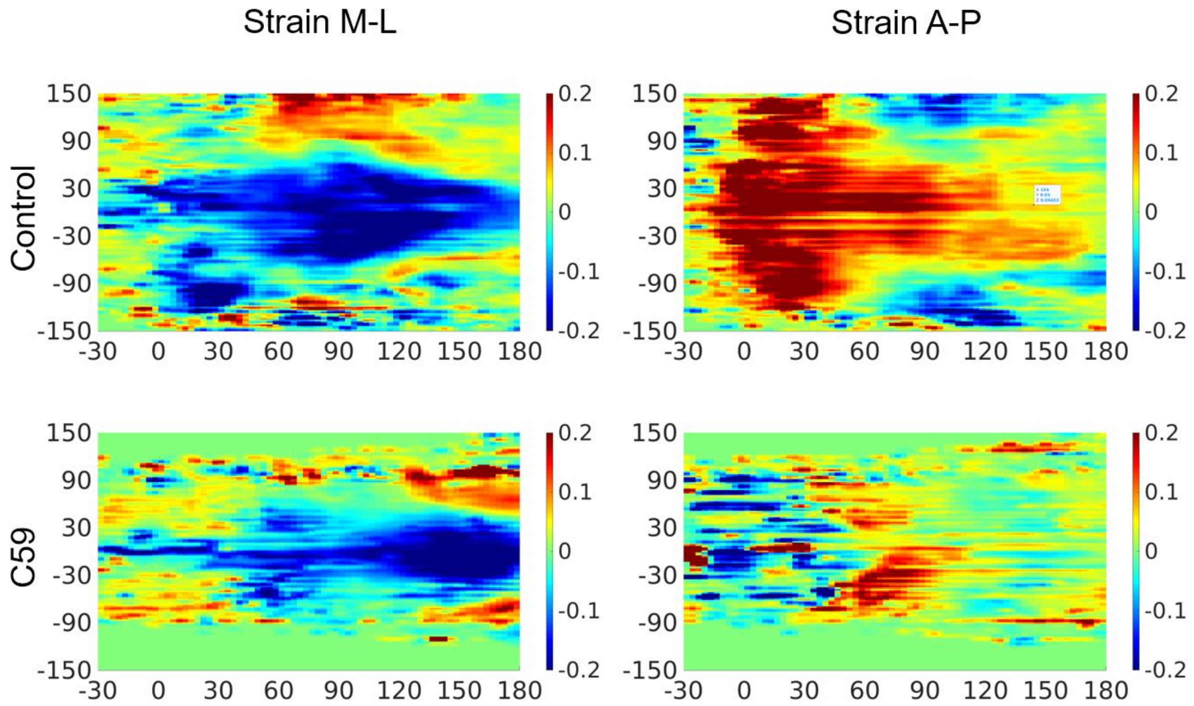

Supplementary Figure 7: The kymograph of Medio-lateral strain (Strain M-L) and Anterior-Posterior strain (Strain A-P) along the equatorial axis for control group and C59 treated group.
